# Unusual Finding of Vertebral Artery Fenestration in Spontaneous Deep Nuclear Hemorrhage

**DOI:** 10.7759/cureus.450

**Published:** 2016-01-06

**Authors:** Karuna Tamrakar, Binod Bhattarai, Sunil Munakomi, Pramod Chaudhary

**Affiliations:** 1 Neurosurgery, College of Medical Sciences, Bharatpur, Nepal; 2 General Surgery, Nobel Medical College

**Keywords:** spontaneous deep nuclear hemorrhage, extracranial, intracranial, vertebral artery fenestration

## Abstract

Vertebral artery fenestration is accidentally detected during angiography or autopsy. Spontaneous deep nuclear hemorrhage in association with vertebral artery fenestration is a very unusual finding in angiography. Such an unusual finding has not been reported in the English literature. Here, we report two cases of spontaneous deep nuclear hemorrhage that presented with features of raised intracranial pressure. Computed tomography revealed a deep nuclear acute bleed in both cases. Digital subtraction angiographic findings were normal other than the presence of a long segment vertebral artery fenestration. Both extracranial and intracranial variations were detected. Although the existence of vascular fenestration in the vertebrobasilar system produces less clinical importance, it may influence the management of cervical and intracranial pathologies to avoid iatrogenic injury.

## Introduction

There are many distinctions in the origin and course of the vertebral artery (VA). This anatomical variation usually affects the surgical procedure. Approximately 1-2% of vertebral artery fenestrations have been accidentally detected at autopsy or on cerebral angiography [1]. Among the 75 cases reported by Hasegawa, et al., 69% were located at the extracranial segment of the VA. Only 20 cases had fenestration at the intracranial segment [2]. Whilst reports on the detection of extracranial fenestration are more frequent than the intracranial segmentation of the VA, both types of fenestration were demonstrated in each of our reported cases [3]. Aneurysmal growth from the fenestrated VA is extremely rare. Additionally, arteriovenous malformation has been explicated in the English literature in association with VA fenestration [4]. However, no report has been specified so far regarding the angiographic detection of a fenestrated VA in a deep nuclear bleed.

## Case presentation

Informed patient consent was obtained from both patients described in this report. No identifying patient information is present in this paper.

### Case 1

**Figure 1:**
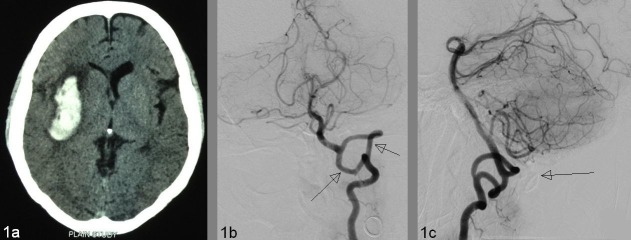
Extracranial vertebral artery fenestration in a 35-year-old hypertensive lady who presented with a right putamen hemorrhage 1A: Acute right putamen hemorrhage in CT scan. 1B: Detection of both widely placed fenestrated segments of VA (arrows) in selective left VA injection (AP view). Length/width of the right limb is 58.7 mm/3.1 mm and the left limb is 62.3 mm/2.9 mm. VA diameters distal to fenestration are 4.0 mm and proximal is 4.3 mm. 1C: Bony landmark of the C1 vertebra (long arrow) in lateral view.

A 35-year-old hypertensive lady presented with the sudden onset of a severe headache, complicated by several episodes of vomiting, and an altered level of consciousness. On examination, the systemic blood pressure (BP) was 220/140 mm of Hg with a Glasgow Coma Scale (GCS) of E3, V4, and M6. Her speech was slurred with right-sided upper motor neuron facial palsy, left hemiparesis of grade 3/5 in the lower extremity, and 2/5 in the upper extremity was present. A CT scan showed a 4.8 x 1.9 cm right putaminal bleed (Figure 1A). Extracranial VA fenestration at the level of C1 and C2 was demonstrated on left VA injection (Figure 1A-1B). Before entering the skull, two limbs of fenestrated VA united to become a normal terminal segment. 

### Case 2

**Figure 2:**
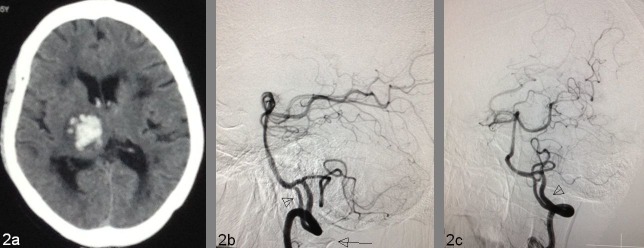
Intracranial vertebral artery fenestration in a 54-year-old man with a right thalamic bleed 2A: Plain CT scan shows a right thalamic bleed. 2B: VA fenestration involving both intracranial (arrowhead) and extracranial segments in lateral view on selective left VA angiography, the long arrow indicating the bony impression of the posterior arch of the C1 vertebra. 2C: Oblique view of VA fenestration (arrowhead). Length/width of the right limb is 20.7 mm/2.2 mm and of the left limb is 33.5 mm/3.1 mm. VA diameters distal to fenestration is 3.4 mm and proximal measurement is 3.9 mm.

A 54-year-old non-hypertensive man presented with a history of the sudden onset of weakness of the right-sided extremities, complicated by headache and nausea. On examination, his BP was 180/110 mm of Hg. GCS was E4, V4, and M6 with right-sided hemiparesis of power grade 1/5. A plain CT scan detected a 2.8 x 2.4 cm acute bleed in the right thalamus and posterior limb of the internal capsule (Figure 2A). On digital subtraction angiogram (DSA), VA fenestration was demonstrated in the selective left vertebral angiography (Figure 2B-2C). 

## Discussion

Fenestrations, duplications, hypoplasias, aplasias, and fetal persistence are the different anatomical variations of the cerebral arteries. The origin is always single in fenestration. Duplication means a division of an artery throughout its course, starting from its origin [5]. Fusion of the duplicated artery has also been explained in the literature. Additional terms have been coined for the fenestration as segmental duplication and for duplication as extreme fenestration [6]. When an embryo develops into the 7-12 mm stage, the VA appears from an anastomosis between the cervical intersegmental arteries in each side. During this stage, when a transient lateral basilar-vertebral anastomosis persists, fenestration of the intracranial portion of the VA results [7]. Lasjaunias, et al., however, elucidated that there are two types of fenestration: an arterial split and true duplication, thereby using fenestration as a generic description of any situation in which there is a double segment of the vertebral artery [8]. Hence, it has been considered as a congenital abnormality rather than being of pathological significance. There are several publications regarding fenestration of the vertebral artery coexisting with cerebral aneurysms [9]. In congenital vascular anomalies like vascular duplications, fenestrations are very frequently associated with other malformations, particularly with cerebral aneurysms and arteriovenous malformations, although corpus callosum agenesis, cervical intervertebral synostosis, multiple block vertebra with partial hemivertebra and severe cervical scoliosis, trigeminal neuralgia, epidermoid cysts, and persistent primitive trigeminal artery have been reported in association with vertebral artery duplication or fenestration [1, 10-12]. However, there has not been any reported case of spontaneous deep nuclear bleed in association with vertebral artery fenestration. Fenestrations of the extracranial course of the vertebral arteries are rare and are usually noted incidentally during angiographic studies or post-mortem examinations [13]. Several theories have been implicated for the development of the fenestration in the intracranial vasculature. Arterial dissection and a thromboembolic event could have been the causes for the basal ganglia bleed that has been majorly supplied by middle cerebral artery perforators [14]. Since the hemodynamic effect in the majority of the developmental vascular anomalies gives rise to the growth of an aneurysm, there is no such clarification to explain the basal ganglia bleed and the fenestrated vertebral artery. It has been stated that vertebral artery duplications or fenestrations were incidental findings with no significant pathologic and clinical consequences.

## Conclusions

Vertebral artery fenestration per se may not predispose to significant vascular consequences. Other than vascular dissection, it can only be the pathogenesis for the development of local vascular disease. The kinking or compressive effect at the origin of fenestration may produce dizziness; however, angiographic detection of vertebral artery fenestration in spontaneous deep nuclear bleed is very rare. Although the existence of vascular fenestration in the vertebrobasilar system produces less surgical importance, it may influence the management of intracranial and cervical pathologies to avoid iatrogenic injury to the vertebral artery.
